# NF-κB—An Important Player in Xenoestrogen Signaling in Immune Cells

**DOI:** 10.3390/cells10071799

**Published:** 2021-07-16

**Authors:** Karolina Nowak, Ewa Jabłońska, Wioletta Ratajczak-Wrona

**Affiliations:** Department of Immunology, Medical University of Bialystok, Waszyngtona 15A, 15-269 Bialystok, Poland; ewa.jablonska@umb.edu.pl (E.J.); wioletta.ratajczak-wrona@umb.edu.pl (W.R.-W.)

**Keywords:** NF-κB, xenoestrogens, xenoestrogen signaling, endocrine disrupting chemicals

## Abstract

The proper functioning of the immune system is critical for an effective defense against pathogenic factors such as bacteria and viruses. All the cellular processes taking place in an organism are strictly regulated by an intracellular network of signaling pathways. In the case of immune cells, the NF-κB pathway is considered the key signaling pathway as it regulates the expression of more than 200 genes. The transcription factor NF-κB is sensitive to exogenous factors, such as xenoestrogens (XEs), which are compounds mimicking the action of endogenous estrogens and are widely distributed in the environment. Moreover, XE-induced modulation of signaling pathways may be crucial for the proper development of the immune system. In this review, we summarize the effects of XEs on the NF-κB signaling pathway. Based on our analysis, we constructed a model of XE-induced signaling in immune cells and found that in most cases XEs activate NF-κB. Our analysis indicated that the indirect impact of XEs on NF-κB in immune cells is related to the modulation of estrogen signaling and other pathways such as MAPK and JAK/STAT. We also summarize the role of these aspects of signaling in the development and further functioning of the immune system in this paper.

## 1. Introduction

In 1986, in the journal *Cell*, Sen and Baltimore described for the first time the transcription factor NF-κB, which is a central element of the signaling pathway and is considered the main regulator that controls the expression of inflammatory mediators in immune cells [1]. NF-κB regulates the immune transcription programs associated with gene encoding, the products that play a key role in the response to bacterial and viral invasions, and are involved in the differentiation and maturation of immune cells as well as the development of lymphatic organs. The expression of NF-κB is regulated at multiple levels and may be influenced by both endogenous and exogenous factors [2,3].

**Figure 1:**
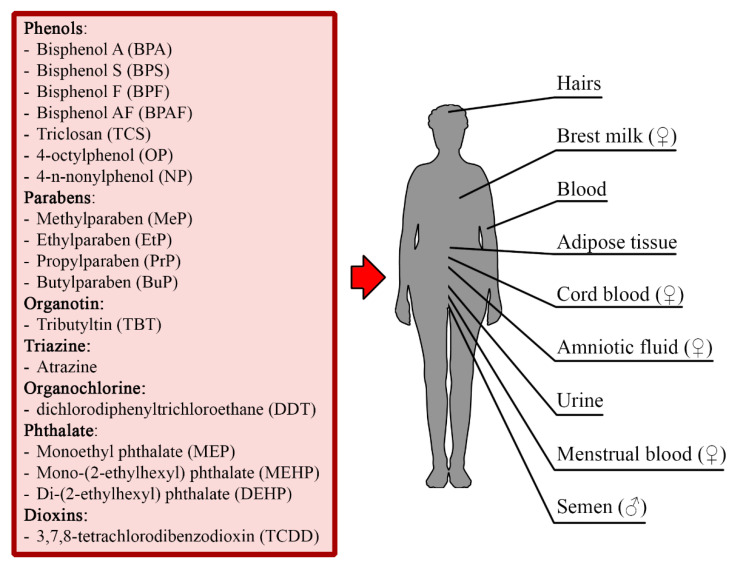
Xenoestrogens in human tissues. Xenoestrogens are absorbed into the human body via digestion, inhalation and transdermal absorption. Compounds were detected in human hairs, breast milk, blood, adipose tissue, cord blood, amniotic fluid, urine, menstrual blood, and semen [13,14,15,16,17,18,19].

Due to the latest trends promoting an ecological and healthy lifestyle, researchers show an increasing interest in the exposure of humans to chemical substances, which are found in food, drugs, cosmetics, plastics, and detergents (Table 1) [4,5,6,7,8,9,10,11,12]. Some ingredients of these products are substances that mimic the action of endogenous estrogens and are collectively known as xenoestrogens (XEs) (Figure 1) [13,14,15,16,17,18,19]. These substances affect the organisms mainly by interacting with their nuclear hormone receptors and modulating the intracellular signaling pathways. The primary targets of XEs in the human body are the elements of the endocrine system. Recently, it was shown that the biological effects resulting from XEs exposure can be observed in all tissues containing the nuclear hormone receptors. This finding suggests that the presence of estrogen receptors (ERs) makes the immune cells potentially sensitive to XEs (Table 2) [20,21,22,23,24,25,26,27,28,29,30,31,32,33,34,35,36,37,38,39,40,41,42]. Moreover, the relationship between XEs and the NF-κB pathway has been confirmed: environmental-origin estrogens modulate NF-κB signaling in immune cells may lead to multidirectional immune disturbance [43].

In this review, we have attempted to answer the question: how do XEs modulate the expression of the NF-κB pathway in immune cells? Through a comprehensive analysis of the available literature data, we assessed the direct effects of XEs on NF-κB, as well as the indirect NF-κB regulation (via the MAPK, PI3K/Akt, and JAK/STAT pathways). We have considered the relationship between NF-κB and estrogen signaling in immune cells exposed to XEs, as well as we discussed the role of the above interactions for development of embryos immune system and their further functioning.

## 2. Xenoestrogens—Environmental Estrogens

In 1991, during the Wingspread Conference in Wisconsin, USA, the public heard for the first time about endocrine disrupting chemicals (EDCs), which are compounds affecting the functioning (in particular, the endocrine system) of living organisms [44]. Depending on the adopted classification criteria, the list of EDCs includes hundreds or even thousands of substances. In addition, new potential EDCs are being identified every day. EDCs exhibit a high degree of heterogeneity in terms of structure and physicochemical properties. For practical reasons, the classification of these compounds into coherent and less numerous groups seems to be crucial for understanding their mechanisms of action. Due to their strong similarity to estrogens, a subgroup called XEs was separated from the large group of EDCs [45,46]. XEs include bisphenols, parabens, dioxins, phenols, and phthalates, which are found in plastic ingredients, internal coats of aluminum cans, detergents, plant-protecting products, and preservatives used for cosmetics, food, and personal care products [4,5,6,7,8,9,10,11,12,47].

**Figure 2:**
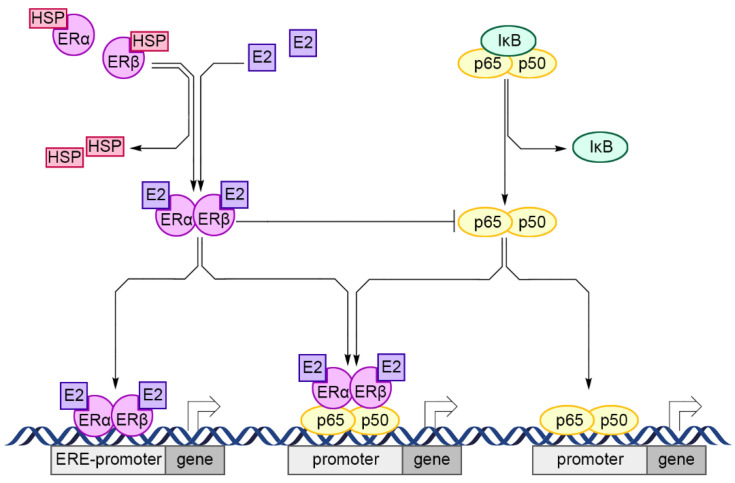
Schematic overview of the ligand-dependent activation of ERs and its interaction with NF-κB pathway in immune cells. HSPs dissociate ERs and allow E2 to bind with them. Free ERs undergo conformational transformations that allow their dimerization. In the nucleus, ERs may bind EREs to interact with other transcription factors. Additionally, ERs suppress NF-κB pathway: ERs may directly bind NF-κB subunits or block NF-κB-binding sites on genes promoter. Abbreviations: E2—estradiol; ERα/β—estrogen receptor α/β; EREs—estrogen response elements; HSP—heap shock protein; NF-κB—nuclear factor κB [48,49,50].

XEs display a complex mechanism of action in cells, but a common point in the action of these compounds is their interaction with nuclear ERs. ERs are localized in the cell cytoplasm in an inactive form, associated with heat shock proteins (e.g., HSP70 and HSP90). The natural ligands for these receptors are the steroid hormones, namely estrone, estriol, and estradiol. These ligands enter the cell, bind with ERs, and dissociate them from HSPs. Subsequently, the dissociated ERs undergo conformational transformations that allow their dimerization. The newly formed ER homo- or heterodimers, which are made of ERα and/or ERβ, can (I) acquire transcriptional activity on their own or (II) by interacting with other transcriptional factors (e.g., NF-κB, SP1, AP-1, and C/EBPβ) (Figure 2). In the nucleus, the activated ERs may bind the estrogen response elements (EREs) present on the promoter of the target gene or regulate the expression of genes without the involvement of EREs [48,49,50]. ERs may also be activated by the activation of transcription factors, which leads to ligand-independent phosphorylation of ERs [49]. Estrogen signaling involves the activation of a fast (seconds or minutes) nongenomic pathway, known as membrane-initiated steroid signaling. Furthermore, stimulation of G protein-bound membrane receptors (GPER, previously known as GPR30) results in immediate changes in the intracellular Ca^2+^ concentration, generation of cAMP and nitric oxide (NO), and activation of phospholipase C and signaling pathways [51,52,53,54].

Research over the last 20 years has confirmed that XEs can adversely affect living organisms, including humans. The effects resulting from exposure to these substances are not just limited to the functioning of the endocrine system, as was previously suspected, but also induce hormonal imbalance and promote the development of obesity. Moreover, XEs are capable of affecting the reproductive system and reducing the ability to conceive healthy offspring [55,56,57]. An alarming discovery is the fact that the effects associated with exposure to XEs may manifest in subsequent generations [58,59]. In recent years, XEs exposure has been linked with impaired memory and learning processes, as well as with Attention-Deficit Hyperactivity Disorder in children [60,61,62].

Furthermore, the list of XE-induced effects includes the disorders that modulate the maturation and functioning of immune cells [43]. XEs have been shown to impair antimicrobial and antiviral responses, and also affect the immunocompetent cells that fight against tumorous cells, thereby indirectly contributing to the progression of neoplastic processes [63,64,65]. Some researchers associate exposure to XEs with the increasing incidence of autoimmune diseases such as thyroid disorders and type I diabetes [66,67,68]. In addition, XEs disrupt the balance between Th1 and Th2 cells, modulate the activity of Th17 cells, and inhibit innate immunity, which indicates its involvement in the development of asthma and allergies [69,70].

The abovementioned disorders result from complex reactions that are mainly related to the malfunction of immune cells. All the intracellular processes are controlled by a network of messenger proteins which are grouped into signaling pathways. Due to their complexity and possible interactions, these signaling pathways create an intracellular signaling network. Among the intracellular pathways in immune cells, NF-κB is especially considered important as it regulates over 200 genes, including those responsible for the production of cytokines, generation of reactive oxygen and nitrogen species, as well as degranulation and maturation of cells. Moreover, the expression of this transcription factor is regulated by both endogenous (e.g., estrogen hormones) and exogenous (e.g., XEs) factors. 

Estrogen-induced interactions between ERs and NF-κB pathway proteins in immune cells lead to several biological reactions, most of which are immunosuppressive. Increased expression of ERα was found to reduce IL-6 production by blocking the NF-κB-binding site on the promoter of the IL-6 gene [71,72,73]. In addition, it was shown that ERβ overexpression inhibits the classical pathway of NF-κB activation, leading to a reduction in LPS-induced production of TNF-α, IL-1β, MCP-1, and IL-6 [74]. Moreover, ERs can directly bind c-Rel and p65 NF-κB, and thus inhibit the activation of the NF-κB transcription factor [49,71,72]. 

## 3. NF-κB Signaling Pathway in Immune Cells

**Figure 3:**
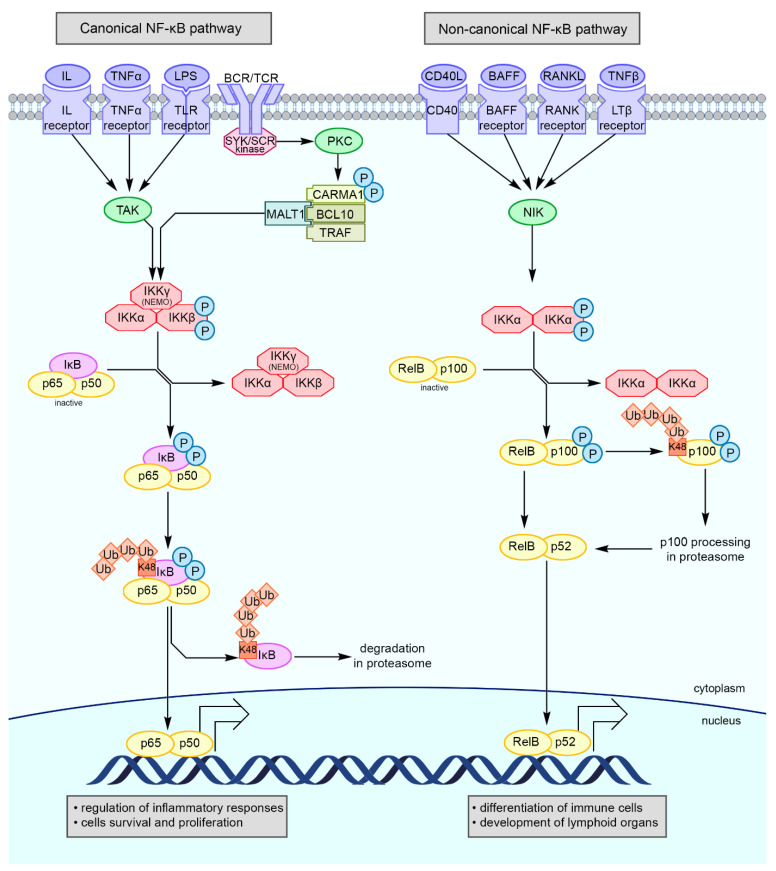
Schematic overview of the canonical (classical) and non-canonical (alternative) NF-κB signaling pathways. Activation of the canonical cascade of NF-κB requires signal transmission via membrane receptors and activation of IKK complex containing NEMO. IKK phosphorylates IκB inhibitor, which is binding NF-κB dimers: p65 and p50. K48-linked polyubiquitination leads to proteasomal degradation of the phosho-IκB, whereas K63-linked polyubiquitin is responsible for stabilizing the membrane receptor signalosome, enable recruitment of downstream adaptors or complexes, and activating kinases. Released NF-κB dimers are translocated into the cell nucleus and regulate transcription of genes. Non-canonical NF-κB pathway is dependent on activation NIK and IKKα complex. The NF-κB dimers remain inactive until IKKα complex phosphorylates p100. Phosphorylation and K48-linked polyubiquitination of p100 cause its proteasome processing which leads to forming p52 subunit. RelB and p52 NF-κB dimers are translocated into the cell nucleus and act as a transcription factor. Abbreviations: BAFF—B-cell activating factor; CD40L—cluster of differentiation 40 ligand; IKK—IκB kinase; IL—interleukin; K48/K63—lysine 48/63; LPS—lipopolysaccharide; LTβ receptor—lymphotoxin β-receptor; NF-κB—nuclear factor κB; NIK—NF-κB-inducing kinase; P—phosphorylation; RANK—receptor activator of nuclear factor kappa-Β; RANKL—receptor activator of nuclear factor kappa-Β ligand; TAKTGF-β-activating kinase; TLR receptor—Toll-like receptor; TNFα/β—tumor necrosis factor α/β; Ub—ubiquitination [75,76,77,78,79,80,81].

The central element of the NF-κB pathway is the homo- or heterodimers composed of two of the following subunits: p65 (also known as RelA), RelB, c-Rel, p50, or p52. Among these, p65/p50 dimer is the most dominant. Before activation, the dimers remain inactive in the cytoplasm of immune cells, for example, bound with IκB inhibitors (Figure 3) [75,76,77,78,79,80,81]. A characteristic feature of NF-κB subunits is the presence of the RHD domain at the N-terminus, which is involved in subunit dimerization and interacts with the IκB inhibitor. Due to the presence of the PEST domain (a region rich in proline, glutamine, serine, and threonine) at the C-terminus of the IκB inhibitor, the transcription factor NF-κB bound with the inhibitor is anchored in the cytoplasm in an inactive form [76,79].

The classical IκB inhibitors (IκBα, IκBβ, and IκBε) bind to the p65 or c-Rel subunit, while the nonclassical ones (IκBζ and Bcl-3) may bind to any of the NF-κB subunits [76,79]. Of these, IκBα is the most common NF-κB inhibitor. The inhibitory function may be performed by IκB-like proteins, which are formed during the proteolysis of the p50 and p52 precursors, known as p105 and p100, respectively [75,79].

NF-κB-dependent genes are transcriptionally controlled by the activation of classical or alternative signal transduction pathways. Despite the differences between them, the two pathways of NF-κB activation may cross each other and should therefore be considered as different axes of the same signaling system.

The classical (canonical) pathway of NF-κB is activated through the enzymatic activity of a protein, composed of IκB kinase (IKK)-α or IKKβ, which binds to the regulatory subunit IKKγ (NEMO). IKK-induced IκB phosphorylation initiates the detachment of inhibitor from dimers, followed by which the inhibitor is ubiquitinated and degraded in the proteasome while the released NF-κB dimers translocate to the cell nucleus. The presence of the RHD domain in NF-κB allows it to acquire the transcriptional activity [76,77,79]. The activation of the NF-κB classical pathway depends on, for example, the stimulation of cytokine receptors, TNF superfamily receptors, pattern recognition receptors, and B cell and T cell receptors [78].

The heterodimers of p52 and RelB are activated through an alternative (noncanonical) pathway, the most important element of which is NF-κB-inducing kinase (NIK). NIK phosphorylates IKKα and triggers the phosphorylation of the p100 precursor. The proteolytic modification of p100 leads to the degradation of the C-terminal IκB-like structures, resulting in the formation of p52, which is translocated to the nucleus along with RelB [75,77,79,80]. 

NF-κB pathways have broad-range competencies in humans including controlling the survival of immune cells, generating inflammatory mediators, and ensuring proper functioning of immune organs. NF-κB is crucial for hematopoiesis and the development of primary and secondary lymphoid tissues, and is activated in thymocytes during positive and negative selection [81,82,83]. RelB plays a key role in the development of the thymus as well as the maturation and functioning of dendritic cells, and its deficiency in humans results in dysmaturity of T and B cells, lack of CD27+ memory B cells, reduced T cell output from the thymus, abnormal clonal expansion of T cell subtypes, and severe T and B cell immunodeficiency [82,84,85,86,87,88]. NF-κB regulates the early development of B cells and survival of naive B cells. Both RelA and c-Rel are involved in the maturation of B cells and control their movement in germinal centers [89,90,91].

The activity of NF-κB is monitored using several techniques which allow evaluating signal transduction at multiple stages of the pathway cascade. Among them, the following are recommended for use in immune cells: Western blot with specific antibodies for monitoring posttranscriptional modification (phosphorylation, acetylation, and ubiquitination) of IκB and NF-κB dimers, and gel-based detection for monitoring changes in protein mobility or changes caused by loss of signal from proteins that were degraded in proteasome. The binding of DNA to the target genes of NF-κB may be tested by electrophoretic mobility shift assay (EMSA), chromatin immunoprecipitation methods, or using the reporter genes as indicators of NF-κB activity at the transcriptional level. Moreover, some techniques enable visualizing the translocation of dimers and their distribution between the cytoplasm and nucleus of cells. It is also recommended to measure the expression of dimers in cytoplasmic and nuclear fractions by Western blot, or using image-based methods in which the translocation of dimers is monitored by antibody staining or fluorescent proteins [92,93,94,95,96,97,98,99,100,101].

## 4. NF-κB as The Target of Xenoestrogens in Immune Cells

### 4.1. Classical Modulation

Both endo- and exogenous substances may affect intracellular processes by binding to membrane or transmembrane receptors. One of the fundamental tasks of immune cells is to recognize and eliminate pathogenic factors, which are receptor-dependent processes. In many cases, these processes are sensitive to bacterial LPS—Toll-like receptors (TLRs). However, research conducted by Pal et al. [102] in rat macrophages exposed to nonylphenol (NP) and LPS did not confirm or deny the involvement of TLR4 in downregulating the expression of NF-κB pathway proteins. Despite the lack of changes in TLR4 expression, NP-exposed cells showed decreased LPS-induced translocation of NF-κB p65 to the nucleus. This effect on intracellular pathways, with the simultaneous lack of involvement of membrane receptors, may possibly result from XEs delving into cells by passive transport. The lipophilicity of these substances is related to their structural similarity to steroid hormones [103]. Thus, XEs may “bypass” the first step of transduction (membrane receptors) and directly interact with the intracellular receptors or pathway proteins inside the cells.

A classical way to modulate the expression of NF-κB is the phosphorylation of IκB inhibitor by IKK. To our knowledge, the influence of XEs on IKK activity in immune cells has not been studied yet. However, the effect of these compounds on IκB expression was already assessed. In their study on murine RAW264.7 cells incubated with bisphenol A (BPA; 10–50 µM), Huang et al. [104] observed intensified degradation of IκB and increased expression of p65 NF-κB in the cell nucleus. Similarly, in RAW264.7 cells exposed to glycidyl-methacrylate (BisGMA), which is an analog of BPA widely used in dentistry, IκB degradation was observed and its intensity was directly proportional to the time of exposure (5–120 min) and concentration of BisGMA (0.1–3 µM) [5]. However, other researchers [105] did not observe any impact of dichlorodiphenyltrichloroethane (DDT), BPA, and 2,3,7,8-tetrachlorodibenzodioxin (TCDD) (1 µM) on IκBα in Jurkat T cells. Moreover, they showed that TCDD and DDT exerted a suppressive effect on NF-κB expression and IL-2 production. Taken together, these results suggest that modulation of IκB may be cell-specific. In RAW264.7 cells, XEs induced the detachment of IκB inhibitor from NF-κB dimers, while in Jurkat T cells, IκBα remained unchanged. As mentioned above, the influence of the time of exposure and concentration and type of XEs on IκB expression cannot be excluded.

In the literature, we can find studies evaluating the expression of NF-κB subunits by Western blot. However, without obtaining information about its posttranscriptional modification, such as by investigating the ratio of expression of the nonphosphorylated and phosphorylated subunits, it is difficult to draw a conclusion about the activation of NF-κB [94]. Most of the available studies have focused on p65 and p50 as common subunits in the canonical pathway. Although it is confirmed that p52, RelB, and c-Rel dimers play a role in the development and maturation of immune cells and organs, their involvement in XE-induced signaling, to our knowledge, has not been investigated.

A study on mice exposed to 200 and 400 mg/kg of atrazine showed increased expression of p65 NF-κB in splenocytes in comparison to cells isolated from animals that were not fed with XEs. Modulation of NF-κB expression by atrazine resulted in enhanced release of reactive oxygen species (ROS) in a dose-dependent manner [106]. In an in vitro study, conducted in our laboratory, Ratajczak-Wrona et al. [107] observed an increase in iNOS-dependent production of NO with a simultaneous increase in the expression of p65 NF-κB in BPA-exposed neutrophils (3–12 μM). The analysis of p65 NF-κB expression in the cytoplasmic and nuclear fractions of neutrophils revealed the differences between the results observed in donors of different sexes. It cannot be ruled out that variations in the level of NF-κB expression in male and female cells may be related to differences in the baseline estrogen concentrations that exacerbate/weaken the BPA-induced effect.

Another technique used experimentally for the evaluation of NF-κB activation is flow cytometry. This method has been used to assess the influence of bisphenols on the development, maturation, and functions of human monocyte-derived dendritic cells. It was shown that expression of the phosphorylated p65 subunit was not changed in cells exposed to bisphenol AF, but increased after simultaneous incubation of cells with LPS and bisphenol AF. This finding suggests that only in activated cells, bisphenol AF may modulate signal transduction via p65 NF-κB [108]. Similar results were noted in RAW264.7 macrophages incubated with LPS and BPA (10 and 50 µM), in which NF-κB-dependent luciferase gene expression was observed to be increased in comparison with nonexposed cells. At the same time, BPA suppressed LPS-induced NF-κB activation (which was still higher compared to the control cells) in a dose-dependent manner [109], whereas a dose-dependent increase of NF-κB-dependent luciferase gene expression was observed in RAW264.7 macrophages exposed to other XEs, namely DDT (0.2, 0.5, or 1 µM) without prior activation of cells by LPS. The activation of NF-κB pathway in response to DDT was confirmed by EMSA. Moreover, the NF-κB activation was associated with intensified production of IL-1β, IL-6, TNFα, and NO [110].

Contradictory results were obtained by Frost et al. [111], who performed Western blot, confocal microscopy, EMSA, and analysis of NF-κB-dependent reporter gene activity. Confocal microscopy analysis showed that translocation of p65 NF-κB subunits was inhibited in the cells exposed to XEs. Moreover, Western blot analysis confirmed the decreased expression of p65 NF-κB (but not p50 NF-κB) in the nuclear fraction of IC-21 macrophages exposed to propanil. Frost et al. [111] demonstrated that exposure of macrophages to propanil reduced the ability of p65/p50 heterodimers and p50/p50 homodimers to bind DNA, the transcriptional activity of NF-κB, and the promoter activity of TNF-α in the regions containing NF-κB-binding sites. Thus, in this complex study, the authors observed that NF-κB activity was suppressed at various stages of the pathway cascade in IC-21 macrophages exposed to propanil.

Literature data indicate that NF-κB is not always the main target of XEs in peripheral blood immune cells (natural killer cells, peripheral blood mononuclear cells (PBMCs), and granulocytes). For instance, Brown and Whalen [112] assessed the expression of ERK1/2, p38 MAPK, NF-κB, and caspase 1 in cells incubated with tributyltin (5, 10, and 25 nM) and observed that the modulation of IL-1β expression was mainly caused by MAPK (ERK1/2 and p38), whereas NF-κB played only a complementary role.

### 4.2. NF-κB in Signaling Network

The signaling proteins in cells are grouped as so-called signaling pathways. The final effect of the signaling cascade is the modulation of gene expression, which enhances or inhibits the regulated processes (e.g., protein synthesis, cell maturation, apoptosis). To ensure the proper functioning of cells, a given effect can be achieved by activating various signaling pathways. The pathway proteins may interact with each other at different levels of the signaling cascade, and by blocking any of the steps in signal transduction, the obstacle can be “bypassed.” In this respect, the NF-κB signaling pathway is no exception. The activity of the NF-κB transcription factor may be affected by proteins from other intracellular pathways, or NF-κB may induce changes in the expression of proteins from other pathways and transcription factors.

Lee and Lim [113] demonstrated that MAPK and PKC interplayed with NF-κB in XE-exposed cells. They observed that the expression of p65 or p50 subunits was increased in the HMC-1 cells exposed to BPA (50 µM). Moreover, in BPA-exposed cells, p38 MAPK expression and PKC translocation were showed. However, the expression of p65 or p50 decreased markedly in the cells simultaneously incubated with BPA and a p38 MAPK inhibitor (SKF86002) or with BPA and PKC inhibitor (staurosporine). Therefore, the authors suggested that BPA-induced activation of NF-κB in HMC-1 cells depends on prior signal transduction via p38 MAPK and PKC. Similar relationships were observed in RBL-2H3 cells exposed to di(2-ethylhexyl)phthalate (DEHP; 100 µM) or BPA (50 µM)—activation of p65 and p50 NF-κB was influenced by the modulation of the signaling cascade at higher levels (PKC and ERK1/2 MAPK) [114,115].

BPA (10–50 µM) induced IκB-dependent activation and translocation of p65 NF-κB into the nucleus of RAW264.7 macrophages as well as increased the expression of other pathways proteins such as ERK1/2, p38 MAPK, JNK, JAK1, JAK2, STAT1, and STAT3. These proteins are important elements in the upstream regulation of NF-κB in immune cells. The presented results suggest that, at least partially, BPA-induced modulation of NF-κB expression depends on the activation of MAPK and JAK/STAT signaling cascade [104]. A similar trend was observed in BPA-exposed THP-1 macrophages. Incubation of these macrophages with ERK1/2 pathway inhibitor (U0126) decreased the expression of IκB and NF-κB as well as inhibited the promoter activity of NF-κB. Based on the obtained results, the researchers confirmed that MAPK represents a higher level of BPA-induced regulation of NF-κB [116]. Another intracellular regulator of NF-κB in immune cells is the PI3K/Akt pathway. Kuan et al. [5] observed increased phosphorylation of Akt in BisGMA-exposed macrophages and suggested that NF-κB expression depends on the activation of the PI3K/Akt pathway.

### 4.3. Crosstalk between NF-κB and Estrogen Signaling

As with estrogens, XEs also elicit a variety of immune cell reactions, some of which have been linked with their direct effects on ERα and ERβ. It has been experimentally confirmed that ERs are involved in the XE-induced modulation of processes including generation of NO and ROS, production of cytokines, as well as degranulation and maturation of cells [116,117,118,119]. Ratajczak-Wrona et al. [119] and Di Pietro et al. [120] showed that, in human neutrophils and PBMCs, BPA regulated the expression of ERα and ERβ in different ways depending on the sex.

ERs may directly modulate the transcription of regulated genes or interact with the NF-κB pathway proteins. For instance, Yoshitake et al. [117] suggested that the inhibition of NO generation in cells following exposure to BPA, NP, and octylphenol was due, at least in part, to the direct effect of these XEs on ERs. On the other hand, it was shown that increased expression of ER dimers reduced the expression of p65 NF-κB in macrophages. Teixeira et al. [121] comprehensively analyzed the influence of BPA, DEHP, and di-n-butyl phthalate (DBP) on ERα- and ERβ-dependent mRNA expression of IκBα, p50 NF-κB, and p65 NF-κB in M1 and M2 macrophages and found that the regulation of intracellular signal transduction in terms of ERs and NF-κB varied depending on the compound tested and the subpopulation of macrophages. Based on the results, the authors indicated that the regulation of IκBα was dependent (at least in part) on ERα in BPA-exposed M1 macrophages as well as in DBP-exposed M2 cells. Moreover, they found that the reduction in the expression of p65 NF-κB in DEHP-stimulated M1 macrophages was influenced by ERβ, while in M2 cells the process was ERα-dependent.

## 5. Xenoestrogen-Induced Signaling in Developing Immune System

Numerous researchers have underlined that exposure to XEs during the early embryonic period may be crucial for the proper development and further functioning of the immune system. Based on their study on mice offspring, Midoro-Horiuti et al. [122] reported that prenatal exposure to BPA may induce asthma. Among the mouse embryonic thymocytes tested, actively differentiating embryonic thymocytes were especially vulnerable to XEs exposure (high expression of T cell receptor and CD5) and died via apoptosis [123]. In a study conducted on a fish model, exposure to XEs led to a concentration-dependent increase in iNOS-dependent production of NO and generation of ROS, as well as to the modulation of cytokine expression [124,125]. 

Similar to the cells of the mature immune system, NF-κB has been recognized as one of the targets of XEs in immune cells, even in embryos. Exposure of *Labeo rohita* larvae in early life stages to BPA increased IκB expression, which explains that XE-induced immunosuppression may result from the suppression of the NF-κB signaling pathway [126]. In fish embryos exposed to BPA, bisphenol S, or bisphenol F, NF-κB was found to be involved in the regulation of IL-1β, IL-6, TNFα, and IFNγ, and with the use of an NF-κB pathway inhibitor, the stimulatory effects on immune-related genes were attenuated [125]. In one of the most recent studies, Liu et al. [127] reported that long noncoding RNA (lncRNA) and their predicted targets (mRNA) should also be considered as the targets of XEs. The authors showed the immunotoxic effects of BPA and its analogs against the primary macrophages of the red common carp (Cyprinus carpio), which were related to changes in the expression of lncRNA and mRNA as well as deregulation of immune-related signaling pathways, including NF-κB, JAK/STAT, B cell receptor, and TLR. However, to our knowledge, no analysis of lncRNA, which may be another factor associated with the mechanism of action of XEs during the development of organisms, has been carried out.

Since we know that the leukocytes of fish express both ERα and ERβ [128], these organisms could be an interesting model for investigating the hypothesis about the crosstalk of ERs and NF-κB in XE-exposed embryos. Moreover, in fish macrophages, ERα but not ERβ signaling was indicated as a regulator of immune effects [129,130]. BPA and its analogs regulate the expression of IL-1β, IL-6, TNFα, and IFNγ via ERα in fish embryos [125]. In contrast to negative crosstalk between NF-κB and ERs observed in mammalian immune cells, the interaction between these two pathways in fish macrophages is positive which, according to researchers’ suggestion, is promoter-specific [130]. 

Due to ethical issues, scientific literature lacks studies about the impact of XEs and their mechanism of action on human embryos. However, researchers have used indirect methods for testing the effects of XEs on the immune system during gestation and their consequences on further functioning. Based on their cohort studies with human participants, Spanier et al. [131] suggested that the critical window of exposure to BPA is early in gestation. They showed the association between high prenatal exposure (at 16 but not 26 weeks of pregnancy) to XEs and the occurrence of wheeze in the child at 6 months of age. In another research, the concentrations of IL-33, IgE, and thymic stromal lymphopoietin in umbilical cord blood and the maternal levels of phthalates, BPA, and perfluoroalkyl were measured. In a Canadian population of pregnant women and their newborns, an association was observed between the concentration of factors, which are integral in the etiology of childhood allergy, and exposure to XEs [132]. However, Donohue et al. [133] did not show any relationship between BPA concentration in maternal urine samples collected during the third trimester of pregnancy and wheeze or asthma in the child. Similarly, Krementsov et al. [134] did not support gestational BPA exposure as a significant contributor to the increased risk of autoimmune diseases (multiple sclerosis); however, researchers observed the modulation of cytokine production by autoreactive T cells in a mouse model. Although the results of another cohort study indicated that prenatal BPA exposure plays a part in the TLR-related innate immune response of neonatal infants, exposure to XEs was not associated with increased risk of infection during early infancy [135].

So far, the mechanism of the abovementioned immune disorders in humans has not been investigated, but based on the results of animal studies, we assume that deregulation of intracellular pathways, such as NF-κB, may be one of the potential elements of their genesis.

## 6. Limitations and Perspectives

Intracellular signal transduction in the immune cells of people exposed to XEs is poorly understood. Among many compounds identified as XEs, only a few have been assessed for their effects on immune cells. Researchers mainly focus on the overall effects of XEs on an organism, but rarely investigate the role of signaling pathways in cells exposed to these compounds. Regarding their effects on the classical pathway of NF-κB activation, it is still unknown whether XEs affect IKK and p52, RelB, and c-Rel NF-κB subunits. To our knowledge, the available literature has no research on the alternative pathway of NF-κB activation in XE-exposed immune cells. Because alternative activating cascade plays a key role in the development and maturation of immune cells, research on this pathway may be extremely valuable in the analysis of possible adverse health effects. 

A proper methodological approach is crucial for assessing XE-induced intracellular signal transduction. Signaling cascade should be evaluated at various levels, using IκB degradation, posttranslational modifications, dimer translocation, and gene regulation as indicators of the activation of NF-κB pathway. In further studies, the principal features of XEs should be considered as additional variables. Data about the role of the sex of the tested subjects, concentration of XEs, and time of XEs exposure in signaling in XE-exposed immune cells are unavailable or limited as these factors have not been thoroughly assessed so far, and so the impact of XEs on the process of intracellular signaling involving NF-κB remains unclear. Moreover, there is a need to experimentally verify the low-dose effects and nonmonotonic dose–response effects of XEs on NF-κB [136]. 

Future research should focus on the molecular mechanism of action of environmental substances in cells, with an aim of understanding the role of XEs in diseases involving abnormal signal transduction. In a study, Bonds and Midoro-Horiuti [137] indicated XEs exposure as one of the factors contributing to the development of autoimmune diseases, asthma, and allergies. Recently, Paciência et al. [138] showed an association between asthma and EDCs exposure in schoolchildren from Portugal. On the other side, Casas and Gascon [70], concluded that the evidence for exposure to phthalates and phenols during the prenatal period and occurrence the respiratory outcomes and allergies are still insufficient. Interestingly, in a few studies, sexual dimorphism in asthma and allergies outcomes were observed. Prenatal exposure to 2,5-dichlorophenol and BPA increased odds of occurrence of asthma among boys [139]. Increased urinary concentration of methylparaben and propylparaben were observed in boys with asthma, but not girls [140]. In a prospective longitudinal study of prenatal and early life, triclosan and paraben exposure were linked with allergic sensitization but only in boys [141]. The possible mechanism of sex-dependent XEs action warrants further exploration with the use extensive research approach: analyzing only one or two hormone receptors will not reveal the interactions responsible for immune-related differences between sex. Future studies examining XE-induced sexually dimorphic effects may be also concentrated on epigenetic reprogramming [142,143]. Moreover, a thorough understanding of the XE-induced mechanism responsible for sex-dependent differences in functioning of immune system is necessary for implementation above findings into the potential clinical use.

As XEs are known to disrupt the functioning of the immune system, the following questions remain to be answered: (I) At what level does the intracellular pathway modulation occur? (II) Is it possible to develop a therapy that involves selective blocking or stimulation of signaling proteins? However, modulation of NF-κB may be extremely challenging due to the ubiquitous presence of the NF-κB pathway proteins in nearly all cells in humans and the direct and indirect regulation of the expression of numerous genes.

## 7. Conclusions

**Figure 4:**
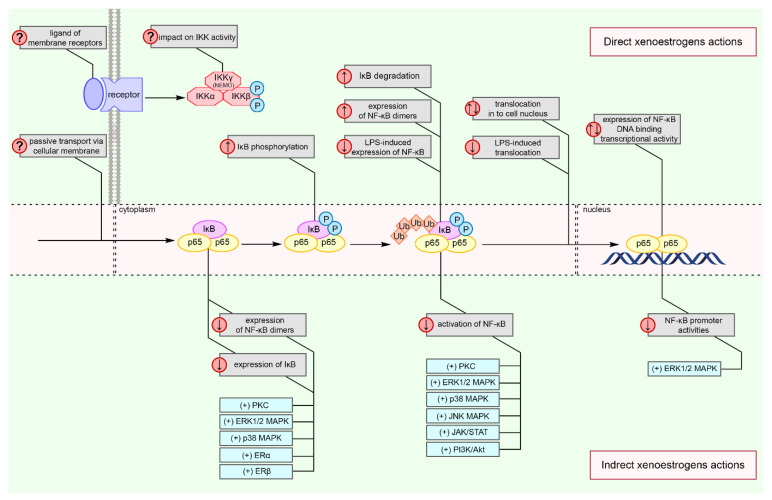
Model of xenoestrogens-induced modulation of NF-κB signaling in immune cells. Xenoestrogens may, directly and indirectly, impact on elements of the NF-κB pathway. Direct effect includes impact on IκB phosphorylation and degradation, NF-κB expression and translocation from cytoplasm to nucleus, as well as a change in transcriptional activity. Xenoestrogens activate numerous intracellular pathway, e.g., ERK1/2 MAPK, JNK MAPK, p38 MAPK, JAK/STAT, PI3K/Akt, PKC as well as ERs signaling, which inhibit NF-κB activation, decrease expression of IκB and NF-κB dimers, and NF-κB promoter activities. Abbreviations: ERK1/2 MAPK—1/2 extracellular signal-regulated kinases pathway; ERα/β—estrogen receptor α/β; IKK—IκB kinase; JAK/STAT—Janus kinases/signal transducer and activator of transcription protein family pathway; JNK MAPK—c-Jun N-terminal kinases pathway; NF-κB—nuclear factor κB; P—phosphorylation; p38 MAPK—p38 mitogen-activated protein kinases pathway; PI3K/Akt—phosphatidylinositol 3-kinase/protein kinase B pathway; PKC—protein kinase C; Ub—ubiquitination; ↑—increase; ↓—decrease; ↑↓—increase or decrease; ?—not tested; (+)—activation [5,102,104,105,107,108,109,110,111,112,113,114,115,117,121].

Our analysis of literature data is the first attempt to determine the effects of XEs on the regulation of NF-κB-dependent intracellular signaling network in immune cells (Figure 4) [5,102,104,105,107,108,109,110,111,112,113,114,115,117,121]. Based on the presented results, we conclude that XEs modulate the classical pathway of NF-κB activation by affecting the degradation of IκB inhibitors, phosphorylation and translocation of dimers, and their transcriptional activity. Modulation of the activity of NF-κB may also result from regulation via p38 MAPK, ERK1/2, PKC, JNK, JAK1, JAK2, STAT1, STAT3, and Akt pathways. Moreover, similar to estrogens, some of the XEs may inhibit proinflammatory reactions by ER-dependent blocking of NF-κB activity.

Most of the available data suggest that the NF-κB signaling cascade is activated by XEs, but some discrepancies are also noted. The contrary results may be due to differences in the mode of action of particular compounds classified as XEs. Although XEs have many similarities, they seem to be nonidentical in structure, effects, and way of action. Moreover, the activation of NF-κB may vary depending on the prior priming of cells (e.g., by LPS) and the tested population (subpopulation) of immune cells.

Researchers suggest that exposure to XEs during the early stage of gestation may affect the proper development of the immune system and its further functioning. Modulation of signal transduction network, in particular the NF-κB pathway, contributes to the development of asthma, allergies, and some autoimmune diseases, in which XE exposure has been indicated as one of the predisposing factors. Therefore, it can be concluded that modulation of the NF-κB pathway may have significant therapeutic potential in the treatment of the abovementioned diseases. 

## Figures and Tables

**Table 1:** Characteristic of xenoestrogens [4,5,6,7,8,9,10,11,12].

Xenoestrogen	CAS No.	Molecular Formula	Source	References
Bisphenol A	80-05-7	C_15_H_16_O_2_	plastics, synthetic resins in baby bottles, children’s toys, food packages, material coating, and medical equipment	[4]
Bisphenol S	80-09-1	C_12_H_10_O_4_S		
Bisphenol F	620-92-8	C_13_H_12_O_2_		
Bisphenol AF	1478-61-1	C_15_H_10_F_6_O_2_		
Bisphenol A glycidyl-methacrylate	1565-94-2	C_29_H_36_O_8_	resin-based dental composite resins and dentin bonding agents	[5]
Triclosan	3380-34-5	C_12_H_7_Cl_3_O_2_	antimicrobial agents in personal care products	[4,6]
4-octylphenol	1806-26-4	C_14_H_22_O	dyeing auxiliaries, surfactant, lubricant additives, pesticide formula, textile printing	[6,7]
4-n-nonylphenol	104-40-5	C_15_H_24_O		
Methylparaben	99-76-3	C_8_H_8_O_3_	antimicrobial preservatives in cosmetics, pharmaceuticals, food commodities and industrial products	[4,8]
Ethylparaben	120-47-8	C_9_H_10_O_3_		
Propylparaben	94-13-3	C_10_H_12_O_3_		
Butylparaben	94-26-8	C_11_H_14_O_3_		
Atrazine	1912-24-9	C_8_H_14_ClN_5_	herbicide	[9]
Dichlorodiphenyltrichloroethane	50-29-3	C_14_H_9_Cl_5_	pesticide	[10]
Monoethyl phthalate	2306-33-4	C_10_H_10_O_4_	plasticizer in polyvinyl chloride (PVC) products, packaging of medical devices, food, and personal care products.	[11]
Mono-(2-ethylhexyl) phthalate	4376-20-9	C_16_H_22_O_4_		
Di-(2-ethylhexyl) phthalate	117-81-7	C_24_H_38_O_4_		
2,3,7,8-tetrachlorodibenzo-p-dioxin	1746-01-6	C_12_H_4_Cl_4_O_2_	pesticide	[12]

**Table 2:** Estrogen receptors in immune cells. Presence of estrogen receptors (nuclear and membrane) in human and animals’ immune cells were confirmed on the protein and RNA level. Abbreviation: GPER—membrane-associated G protein-coupled estrogen receptor, ERRβ—estrogen receptor-related β, ERs(α/β)—estrogen receptors (α/β), mRNA—messenger RNA, RT-PCR—real-time PCR [20,21,22,23,24,25,26,27,28,29,30,31,32,33,34,35,36,37,38,39,40,41,42,43].

Cell Type.	Estrogen Receptor	Material	Subject	Method	References
B cell	ERα; ERβ	Protein	mice	Flow cytometry	[20]
B cell	ERα (46 kDa, 66 kDa); ERβ (56 kDa)	Protein	human	Flow cytometry	[21]
B cell CD19^+^	ERα (low); ERβ (high)	mRNA	premenopausal female, postmenopausal female, male	RT-PCR	[22]
B cell precursors	ERα; ERβ	mRNA	mice	RT-PCR	[23]
Basophilic leukemia cell line RBL-2H3	ERα; lack of ERβ	mRNA	rat cell line	RT-PCR	[24]
Dendritic cell	ERα; ERβ	mRNA	mice	RT-PCR	[25]
Dendritic cell	GPER	Protein/mRNA	human	Western blot/RT-PCR	[26]
Dendritic cell	ERα	RNA	mice	RT-PCR	[27]
Eosinophils	GPER	Protein/mRNA	human	Flow cytometry/Immunochemistry/RT-PCR	[28]
Eosinophils	GPER	Protein/mRNA	human	Western blot/RT-PCR	[26]
Endometrial neutrophils	lack of ERα	Protein	female	Immunocytochemistry	[29]
Macrophages CD68+	ERRβ	Protein	human	Immunocytochemistry	[30]
Mast cell	ERs	Protein	human	Immunocytochemistry	[31]
Mast cell	ERs	Protein	human	Immunocytochemistry	[32]
Mast cell	ERs	Protein	human	Immunocytochemistry	[33]
Mast cell line HMC-1	ERα; lack of ERβ	mRNA	human cell line	RT-PCR	[24]
Monocytes	ERα; ERβ	mRNA	human	RT-PCR	[34]
Monocytes	ERα (low); ERβ (low)	mRNA	premenopausal female	RT-PCR	[22]
Monocytes	ERα (high); ERβ (low)	mRNA	postmenopausal female, male	RT-PCR	[22]
Monocytes	GPER	Protein/mRNA	human	Western blot/RT-PCR	[26]
Natural killer	ERα; ERβ	Protein	mice	Immunocytochemistry	[35]
Natural killers	ERα (46 kDa); ERβ (56 kDa)	Protein	human	Flow cytometry	[21]
Natural killer	ERα (66 kDa); ERβ (56 kDa)	Protein	human	Western blot	[36]
Neutrophils	ERα; lack of ERβ	mRNA	human	RT-PCR	[34]
Neutrophils	ERα (67 kDa); ERβ (56 kDa)	Protein	human	Western blot	[37]
Neutrophils	ERβ	Protein	cow	Flow cytometry/Western blot	[38]
Neutrophils	GPER	Protein/mRNA	human	Western blot/RT-PCR	[28]
Neutrophil like HL-60	ERα; ERβ; GPER	Protein	human cell line	Western blot/Immunocytochemistry	[39]
Primary synovial macrophages	ERα; ERβ	Protein/mRNA	human	Immunocytochemistry/RT-PCR	[40]
T cell	ERα; ERβ	RNA	female, male	RT-PCR	[41]
T cell CD4^+^	ERα (high); ERβ (low)	mRNA	premenopausal female, postmenopausal female, male	RT-PCR	[22]
T cell CD4^+^	ERα	RNA	mice	RT-PCR	[27]
T cell CD8^+^	ERα (low); ERβ (low)	mRNA	premenopausal female, postmenopausal female, male	RT-PCR	[22]
T cell	ERα (46 kDa); ERβ (56 kDa)	Protein	human	Flow cytometry	[21]
Uterine natural killers cells CD56+	ERβ1; ERβcx/β2	mRNA	mice	RT-PCR	[42]
Uterine natural killers cells CD56+	ERRβ	Protein	human	Immunocytochemistry	[30]

## Data Availability

Data sharing is not applicable to this article.
